# Pulmonary surfactant coating of multi-walled carbon nanotubes (MWCNTs) influences their oxidative and pro-inflammatory potential in vitro

**DOI:** 10.1186/1743-8977-9-17

**Published:** 2012-05-24

**Authors:** Michael Gasser, Peter Wick, Martin JD Clift, Fabian Blank, Liliane Diener, Bing Yan, Peter Gehr, Harald F Krug, Barbara Rothen-Rutishauser

**Affiliations:** 1 Adolphe Merkle Institute, University of Fribourg, Marly, Switzerland; 2 Empa, Swiss Federal Laboratories for Materials Science and Technology, St. Gallen, Switzerland; 3 Respiratory Medicine, Department of Clinical Research, Inselspital University Hospital, University of Bern, Bern, Switzerland; 4 Department of Chemical Biology and Therapeutics, St. Jude Children’s Research Hospital, Memphis, TN, USA; 5 School of Chemistry and Chemical Engineering, Shandong University, Jinan, China; 6 Institute of Anatomy, University of Bern, Bern, Switzerland

**Keywords:** Multi-walled carbon nanotubes (MWCNTs), Pulmonary surfactant (Curosurf), Macrophages, Epithelial cells, Dendritic cells, Triple cell co-culture, Pro-inflammatory and oxidative reactions

## Abstract

**Background:**

Increasing concern has been expressed regarding the potential adverse health effects that may be associated with human exposure to inhaled multi-walled carbon nanotubes (MWCNTs). Thus it is imperative that an understanding as to the underlying mechanisms and the identification of the key factors involved in adverse effects are gained. In the alveoli, MWCNTs first interact with the pulmonary surfactant. At this interface, proteins and lipids of the pulmonary surfactant bind to MWCNTs, affecting their surface characteristics. Aim of the present study was to investigate if the pre-coating of MWCNTs with pulmonary surfactant has an influence on potential adverse effects, upon both (i) human monocyte derived macrophages (MDM) monocultures, and (ii) a sophisticated in vitro model of the human epithelial airway barrier. Both in vitro systems were exposed to MWCNTs either pre-coated with a porcine pulmonary surfactant (Curosurf) or not. The effect of MWCNTs surface charge was also investigated in terms of amino (−NH_2_) and carboxyl (−COOH) surface modifications.

**Results:**

Pre-coating of MWCNTs with Curosurf affects their oxidative potential by increasing the reactive oxygen species levels and decreasing intracellular glutathione depletion in MDM as well as decreases the release of Tumour necrosis factor alpha (TNF-α). In addition, an induction of apoptosis was observed after exposure to Curosurf pre-coated MWCNTs. In triple cell-co cultures the release of Interleukin-8 (IL-8) was increased after exposure to Curosurf pre-coated MWCNTs. Effects of the MWCNTs functionalizations were minor in both MDM and triple cell co-cultures.

**Conclusions:**

The present study clearly indicates that the pre-coating of MWCNTs with pulmonary surfactant more than the functionalization of the tubes is a key factor in determining their ability to cause oxidative stress, cytokine/chemokine release and apoptosis. Thus the coating of nano-objects with pulmonary surfactant should be considered for future lung in vitro risk assessment studies.

## Background

The ever-developing industry of nanotechnology the last two decades has culminated in a plethora of new nano-objects (defined as material with one, two or three external dimensions in the nanoscale) which are being used within a variety of consumer and industrial applications. Among the most prominent nano-objects are carbon nanotubes (CNTs); hollow nanofibres formed from carbon [1]. Based on their structure CNTs are classified as either, single-wall carbon nanotubes (SWCNTs), which comprise a single layer of carbon atoms, or multi-wall carbon nanotubes (MWCNTs), comprising of multiple concentric tubes [2]. Structural and mechanical characteristics such as an extreme strength, stiffness and robustness [3] make CNTs interesting for the use in an infinite number of applications such as sporting goods, automobile products or household items. Additionally, CNTs hold great promise for application within medicine, particularly as a tool in therapeutics and diagnostics [4]. With their increasing number of applications, CNT emissions into the environment and human exposure may increase. Mainly CNT production, processing and disposal may be hazardous for humans [5]. Moreover during the use of CNT containing products, CNTs may be released into the environment as for instance from abrasion or degradation of CNT containing products. Possible portals by which CNTs may enter the human body include the skin, the gastro-intestinal tract and injection (nanomedicine). However, it is well accepted from previous research using nano-sized particles [6] and CNTs [7,8] that inhalation is the primary exposure route to the human body if CNTs are released into the environmental air.

Concerns about the safety of CNTs have been raised for a number of different reasons [3,9,10] (i) due to their small aerodynamic diameter CNTs are hypothesised to reach the lower respiratory tract, (ii) CNTs possess, like other nano-objects, a high surface to mass ratio, thus a large surface can interact with the biological surroundings, and (iii) some CNTs which are fibre shaped may (if structured dimensions are similar) behave like asbestos, or other pathogenic fibres which are toxic due to their needle-like shape. Moreover (iv), numerous in vivo studies (e.g.) [11-13] have shown different types of MWCNTs to remain in the lung for up to several months after deposition indicating the potential for prolonged biopersistence.

Recently the potential adverse effects of CNTs have been studied on various biological systems, using different exposure methods both in vivo and in vitro [14,15]. Despite the unrealistically high doses which have been used within some of the previous studies (e.g.) [16], it is known that subpleural fibrosis [17], granuloma formation [10] and mesothelioma [16] similar to the effects of crocidolite asbestos fibres, can appear after in vivo exposures to mainly straight, stiff and extremely long CNTs.

Observed adverse effects have further been explained by the oxidative stress paradigm [18]. In numerous studies (e.g.) [19-21] an increased oxidative stress response in vivo and in vitro has been reported causing a subsequent (pro-) inflammatory reaction after exposures to both straight and tangled CNTs.

Although numerous studies address the adverse potential of CNTs, their comparability is often limited and results are contradictory. Explanations for these discrepancies include differences in administered dose, the physico-chemical characteristics (e.g. agglomeration/aggregation state, metal impurities, stiffness, length) of the CNTs studied, the exposure method of CNTs, or differences in the biological system employed [15,22,23]. Thus conditions/characteristics have to be manipulated systematically in order to identify key factors for their potential (adverse) biological effects. A promising way to modify the properties of CNTs is the functionalization of the surface [24,25]. Functionalization of CNTs can be used to promote the binding of specific biomolecules (such as siRNA) [26] but also to improve their biocompatibility. The adverse effect potential of CNTs can be significantly driven by the particular (surface) modification employed [22] whereas studies have shown both, increases and decreases in toxicity after exposures to CNTs with different surface functionalizations [27,28]. Functionalization can further affect the CNTs dispersity which can have subsequent consequences on their cell uptake and agglomeration in tissue [29].

It is not only artificial surface modifications that play a role in regards to the potential adverse effects of CNTs. The surface characteristics of MWCNTs may also be modified by the adsorption of biomolecules following inhalation, and the subsequent interaction with the lung. Specifically, an initial coating of the MWCNTs will take place when they interact with pulmonary surfactant which is mainly produced by epithelial type II cells and which is located at the air-liquid interface. Surfactant consists 85-90% of phospholipids [30], the specific surfactant proteins (SP) -A, -B, -C, and -D (~10%) and its main function is the reduction of the alveolar surface tension and keeping the gas exchange surface at optimal size during the movements of breathing [31]. Thus, during deposition, surfactant or surfactant components will bind to the surface of MWCNTs [32,33]. Previously, this initial coating has not been sufficiently considered in respect to in vitro lung toxicity studies. The modulation of the adverse potential from surfactant binding is mainly described for microparticles [34], however to the best of our knowledge, not for CNTs and other nano-objects. After inhaled CNTs are coated with pulmonary surfactant, they may be displaced into the aqueous hypophase [35-37] and come in contact with cells of the immune system such as macrophages and dendritic cells, which may engulf the MWCNTs and clear them from this area of the lung [38]. Also epithelial type I cells can interact with CNTs, as these cells mainly cover the alveolar surface [31].

### Objectives of the study and methodological approach

The primary aim of this study therefore, was to investigate how a pre-coating of MWCNTs with pulmonary surfactant may affect their potential adverse effects on cells of the air blood tissue barrier in vitro. In order to simulate the pulmonary surfactant coating, MWCNTs were pre-coated with Curosurf, a well characterized natural porcine surfactant preparation [39-41].

Monocyte derived macrophage (MDM) monocultures as well as a sophisticated 3D in vitro triple cell co-culture model of the airway epithelial barrier [42] were exposed to the MWCNTs either pre-coated with surfactant or not. MWCNTs were initially compared to their potential to affect cell viability. Subsequently, in order to study an early oxidative reaction, reactive oxygen species (ROS) was quantified in MDM. For the characterization of a prolonged oxidative stress response the intracellular antioxidant glutathione was quantified. The release of the cytokine TNF-α, as well as the chemokine IL-8 was quantified in order to study their possible (pro-) inflammatory effects.

The secondary aim was to investigate the influence of the surface charge on the MWCNTs potential adverse effects. Therefore, non-functionalized (“pristine” or “P-MWCNTs”), carboxyl (“MWCNT-COOH”) and amino (“MWCNT-NH_2”_) functionalized MWCNTs were used in the different exposures. In order to study a concentration dependence of potential adverse effects, 2–3 different concentrations (depending on the endpoint, see methods section for details) were applied. Eventually the different conditions (Curosurf pre-coating, functionalization, concentration) were statistically compared.

## Results

### MWCNT characterization

Different MWCNT dispersions were fully characterized prior to the exposures. A detailed overview is given in Additional file 1: Table S1.

### Cell morphology and intracellular localisation of MWCNTs in MDM

**Figure 1:**
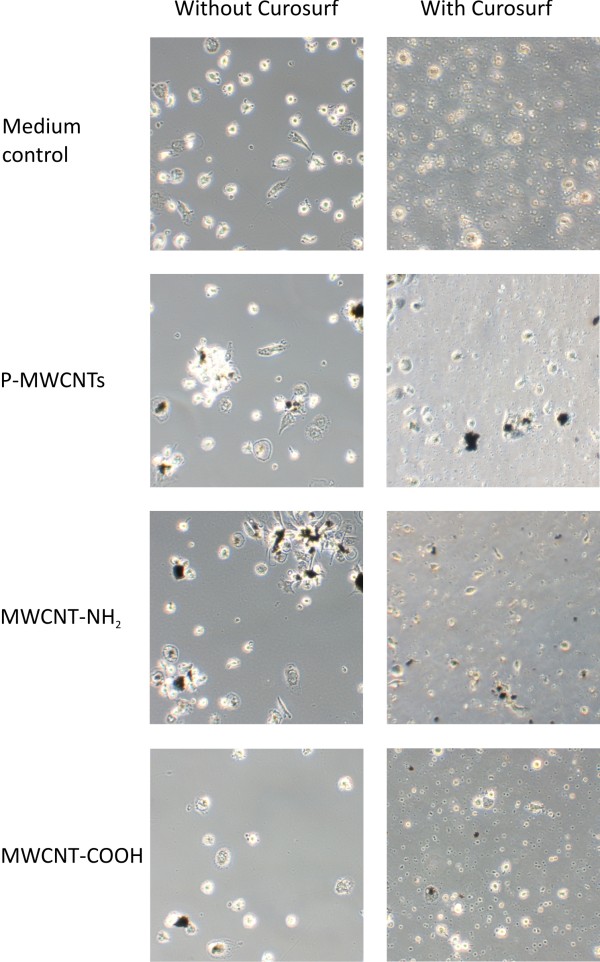
**Light micrographs of MDM.** Top images show MDM in medium and in medium containing 3% Curosurf respectively (MWCNT free controls). For all other conditions MDM were exposed to different functionalized and Curosurf pre-coated MWCNTs (30 μg/ml) for 24 h. A 20x magnification was used.

**Figure 2:**
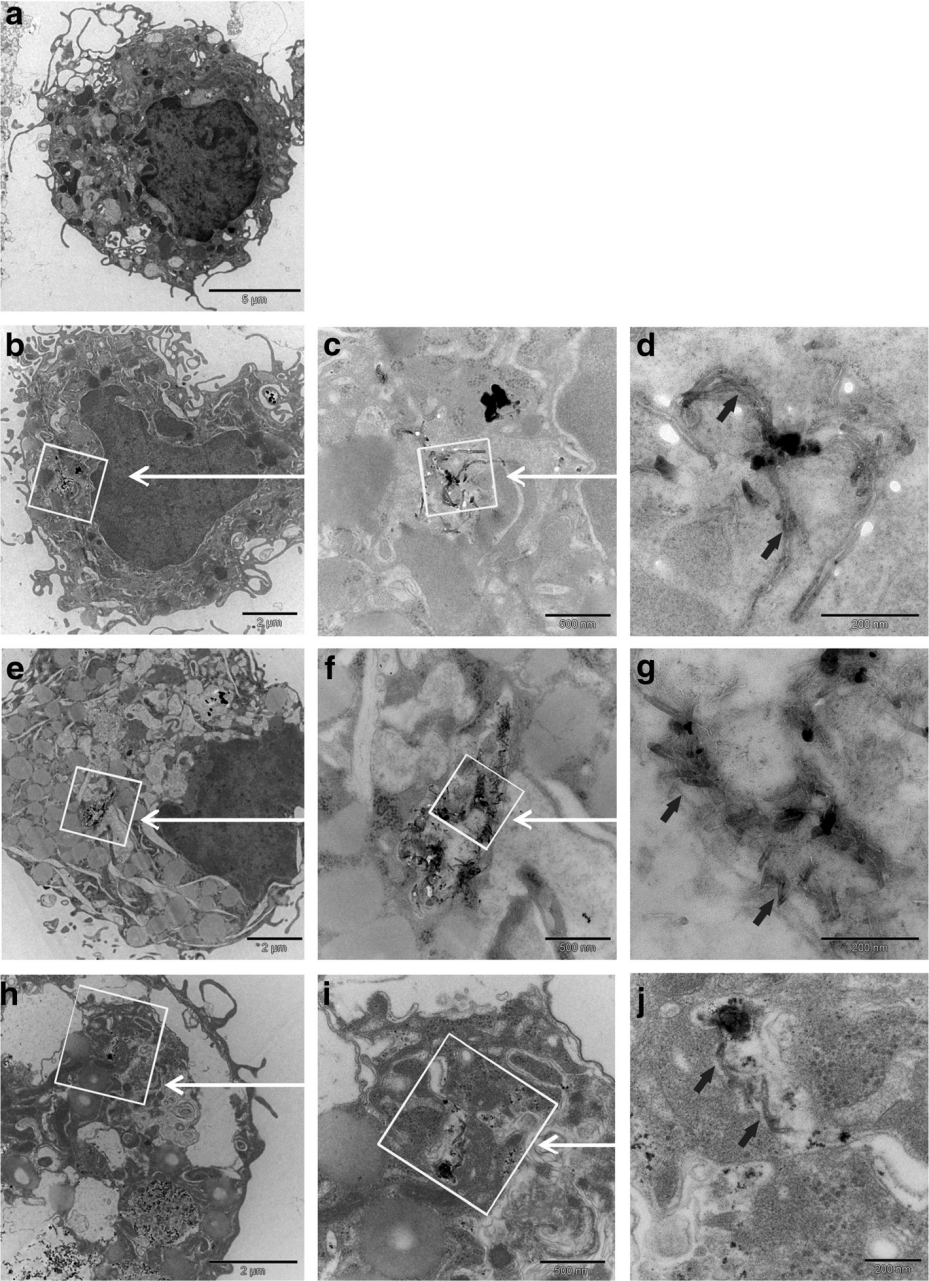
**Transmission electron micrographs of MDM.** MDM were exposed for 24 h to MWCNTs (30 μg/ml) under different conditions (Curosurf pre-coating, functionalization) **a**.: Untreated control. **b.-d**.: MDM exposed to uncoated P-MWCNTs. P-MWCNT agglomerates are visible at low magnification (**b**); high magnification micrographs (**c, d**) reveal individual P-MWCNTs (black arrows). **e**.: MDM exposed to Curosurf pre-coated P-MWCNTs at low magnification; **f**, **g**: higher magnifications with black arrows indicating individual MWCNTs in “bird's-nest”-like arrangements. **h.-j**. MDM exposed to MWCNT-COOH (without Curosurf pre-coating); arrows indicate single MWCNT-COOH. Scalebar is 5 μm for **a**., 2 μm for **b**., **e**. and **h**., 500 nm for **c**., **f**. and **i**., and 200 nm for **d**., **g**. and **j**.

The morphology of the majority of the cells was not impaired after exposures of MDM to different functionalized and +/− Curosurf pre-coated MWCNTs (Figures 1 and 2). Only a small number of cells showed indications (e.g. increased cell volume) for apoptosis after different MWCNT exposures. Larger bundles of tubes could be observed attached on the cell surface and inside cells, but also free in the medium after 24 h. Transmission electron microscopy (TEM) showed smaller bundles, as well as single tubes to be present inside MDM after 24 h of exposure (Figure 2). MWCNTs were found inside MDM both free in the cytoplasm and also in vesicles (Figures 2B- 2J). Curosurf pre-coated MWCNTs were often found inside MDM in larger “bird's-nest”-like arrangements (Figures 2E- 2G). MWCNT-COOH, which were better dispersed than P-MWCNTs, were also localized intracellular by TEM (Figure 2H- 2J).

### Potential adverse effects in MDM monocultures

A variety of conditions such as concentrations, Curosurf pre-coating, and functionalizations were first measured in MDM monoculturures for different endpoints (cytotoxicity, oxidative stress, pro-inflammatory markers and cell viability). From these data a set of conditions were then chosen for the triple cell co-cultures. An overview of the statistical analyses from MDM monocultures as well as triple cell-co cultures can be found in the Additional file 2.

### Cytotoxicity

**Figure 3:**
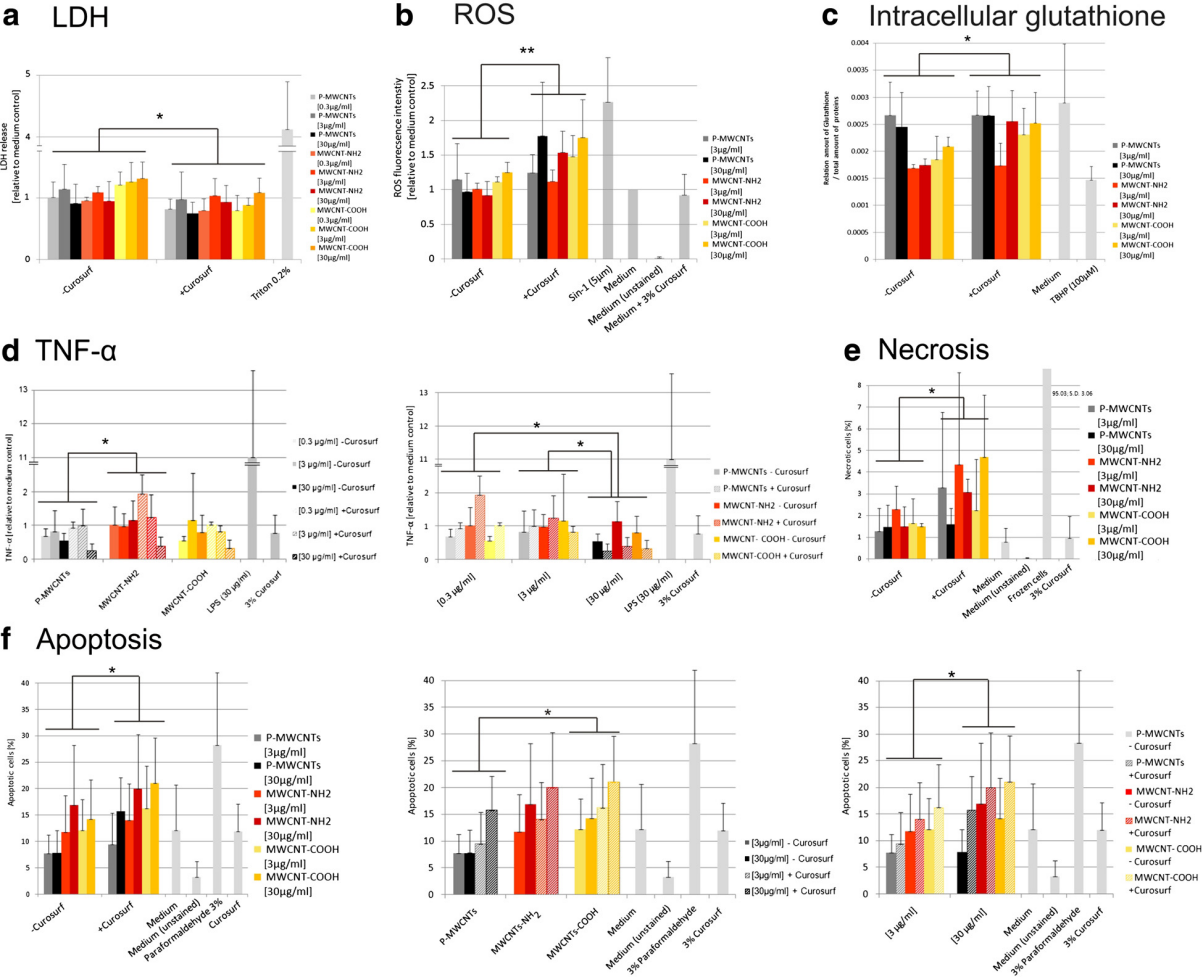
**Endpoints measured in MDM.** Several endpoints in MDM were analysed after exposure to different functionalized and pre-coated MWCNTs. **a**. Cytotoxicity (lactate dehydrogenase release), **b**. Reactive oxygen species, **c**. Intracellular glutathione, **d**. Tumor necrosis factor-α. (For an optimized representation of group comparisons the same data are visualized in two diagrams. Values are shown relative to the medium control which corresponds to a mean concentration of 87.1 pg/ml), **e**. Necrosis, **f**. Apoptosis (For an optimized representation of group comparisons the same data are visualized in three diagrams.). Endpoints were assessed after 24 h exposures apart from ROS (B), which was assessed after 1 h. Experiments have been performed in 3 to 5 repetitions. Data shows mean values ± standard deviation (SD). Different groups were compared by ANOVA and Bonferroni *post hoc* tests (* = *p <* 0.05, ** = *p <* 0.01).

The cell integrity was investigated by the quantification of the total lactate dehydrogenase (LDH) release. Values from uncoated MWCNT-NH_2_ were increased up to 30% (for 30 μg/ml) (Figure 3A). LDH concentrations for all uncoated MWCNTs were found to be slightly higher (*p <* 0.05) (Figure 3A) than for Curosurf pre-coated MWCNTs in MDM exposures.

In a control experiment the binding of the LDH enzyme to the surface of the MWCNTs was not found to interfere with the LDH assay, and thus no false positive/negative toxicity was observed (data not shown).

### Oxidative stress response

For assessing the oxidative potential of MWCNTs, ROS were measured by flow cytometry after 1 h using the fluorescent marker H_2_DCF-DA. A significant increase in ROS (*p <* 0.01) was observed from the Curosurf pre-coated MWCNTs (Figure 3B). ROS levels for uncoated MWCNTs were not significantly above the base levels. Any significant increase in ROS was measured for different cell free MWCNT dispersions (measured by fluorescence spectrophotometry, data not shown).

Glutathione, an intracellular antioxidant, which helps to prevent damage to cellular components caused by ROS [43], was quantified following 24 h exposure of MDM. The intracellular glutathione content was decreased after exposure to functionalized MWCNTs which were not pre-coated (*p <* 0.05) (Figure 3C). No effects were observed after exposure with Curosurf pre-coated MWCNTs, except for a decrease of amino functionalized MWCNTs at 3 μg/ml.

### TNF-α release

Concentrations of the pro-inflammatory cytokine TNF-α were assessed in the cell supernatant via an enzyme linked immunosorbant assay (ELISA) following 24 h exposure. Independent of the MWCNTs functionalization the Curosurf pre-coating caused a decrease in the TNF-α release for the 30 μg/ml exposures. This resulted in a significant (*p <* 0.05) decrease of 30 μg/ml exposures compared to lower concentrations (Figure 3D) and a significant interaction of the independent variables “Concentration” and “Pre-coating” (Additional file 2). To ensure that the decrease in TNF-α release was caused by Curosurf pre-coated tubes and not from free surfactant, the cells were exposed to a control containing cell culture medium and 3% Curosurf (see Methods section). TNF-α values for the free Curosurf were above the values of exposures containing Curosurf pre-coated MWCNTs (Figure 3D). This indicates a stronger effect (on the decrease in TNF-α release) from exposures to Curosurf pre-coated MWCNTs rather than from free Curosurf.

TNF-α release in MDM was further dependent upon the functionalization (*p <* 0.05) (Figure 3D), where higher values were measured after MWCNT-NH_2_ exposures.

In additional cell free control experiments the binding of TNF-α cytokines to free MWCNTs and to Curosurf was investigated by ELISA. No decrease of TNF-α levels was detected after the addition of either Curosurf or MWCNTs to a defined TNF-α concentration (data not shown).

### Cell viability

Apoptotic and necrotic cells were assessed by using an Annexin V staining kit via flow cytometry. Necrosis was observed to be significantly dependent on the Curosurf pre-coating (*p <* 0.05) (Figure 3E). However, the fraction of total necrotic cells was never above 5% for the different conditions studied.

Apoptosis was observed to be significantly dependent on the Curosurf pre-coating (*p <* 0.05). Values for Curosurf pre-coated MWCNTs were increased up to 20% (of the total cell number). Significantly (*p <* 0.05) more apoptotic cells were found after exposures to MWCNT-COOH compared to P-MWCNT exposures.

### Potential adverse effects of MWCNTs on triple cell co-cultures

Due to its higher complexity the triple cell co-culture model could only be used with a restricted set of experimental conditions. In MDM effects from the Curosurf pre-coating were especially pronounced for MWCNT-COOH. Thus in triple cell co-cultures only these tubes were then used for Curosurf pre-coating.

In contrast to MDM monocultures, triple cell co-cultures were grown on transwell membrane inserts. Therefore biochemical endpoint analyses were performed upon supernatants taken from below (“lower well”) and above (“upper well”) the microporous membrane. Data from the lower well thus mainly represent the reaction of monocyte-derived dendritic cells (MDDC), whereas results from the upper well mainly represent the reaction of epithelial cells and MDM (see Method section for details).

### Cytotoxicity

**Figure 4:**
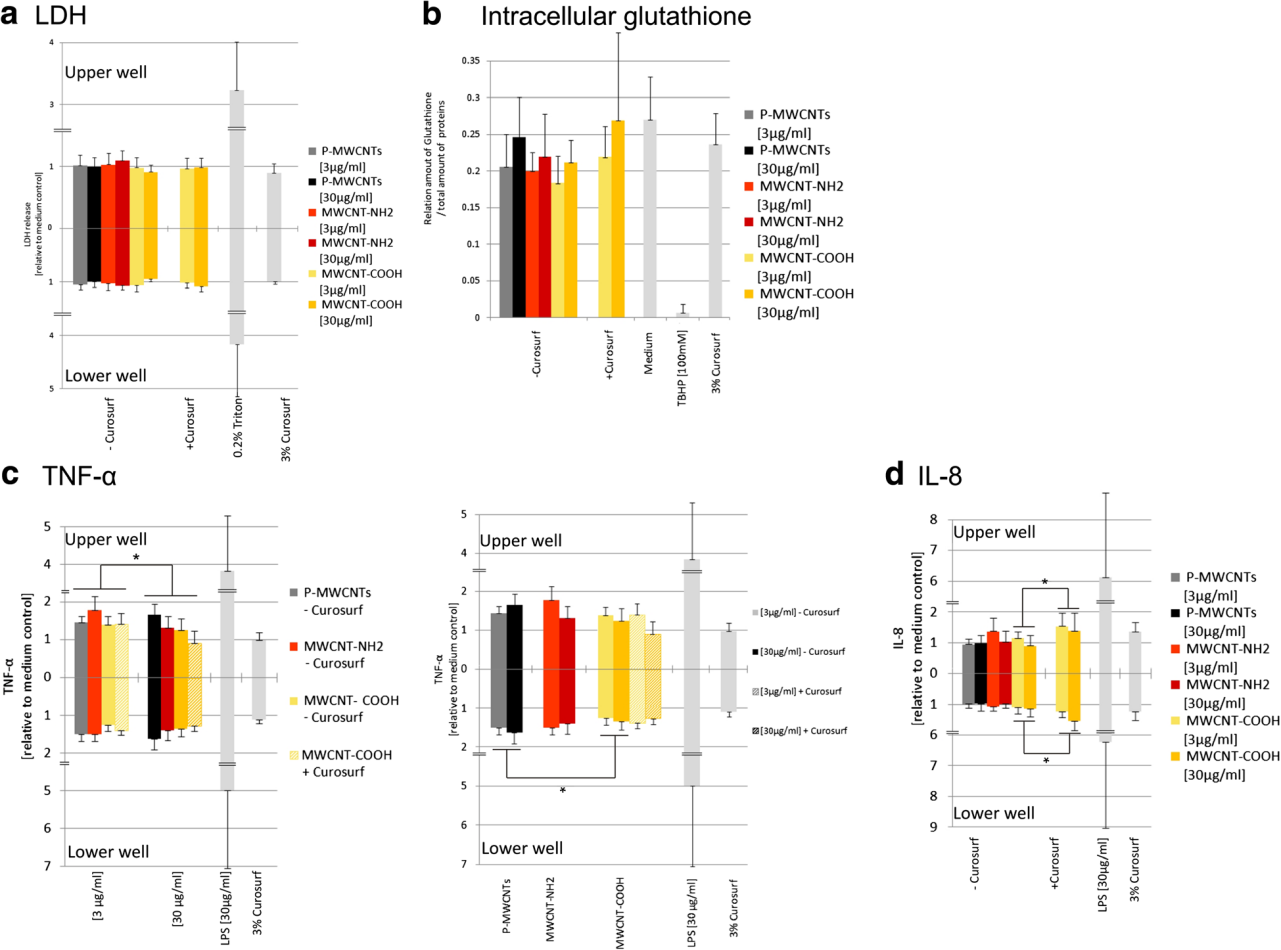
**Endpoints measured in triple cell co-cultures.** Triple cell co-cultures consisting of MDM, MDDCs and 16HBE14o cells were exposed to different functionalized and pre-coated MWCNTs. Endpoints were assessed after 24 h exposures. **a**. Cytotoxicity (lactate dehydrogenase release), **b**. Intracellular glutathione, **c**. Tumour necrosis factor-α (for an optimized representation of group comparisons the same data are visualized in two diagrams. Values are shown relative to the medium control which corresponds to a mean concentration of 249 pg/ml for the upper well and 201 pg/ml for the lower well), **d**. Interleukin-8 (values are shown relative to the medium control which corresponds to a mean concentration of 1.39 ng/ml for the upper well and 1.37 ng/ml for the lower well). Experiments have been performed in 5 repetitions. Bars represent mean values ± SD. Different groups were compared by ANOVA and Bonferroni *post hoc* tests (* = *p <* 0.05).

Cytotoxicity values were not significantly above the baseline levels for all MWCNTs tested (Figure 4A).

### Intracellular glutathione content

No statistically significant effect was found for a specific condition (Figure 4B). Only a tendency for a decrease in intracellular glutathione after 24 h exposure to uncoated MWCNTs was observed. All values of uncoated MWCNT exposures were (up to 30%) below the medium control, whereas the highest concentration of Curosurf pre-coated MWCNT-COOH was within the range of the medium control.

### TNF-α and IL-8 release

TNF-α release was significantly (*p <* 0.05) lower for 30 μg/ml exposures compared to 3 μg/ml exposures (Figure 4C) in the upper well (with the lowest concentrations for Curosurf pre-coated MWCNT-COOH). No concentration dependent effect was measured in the lower well. However, in the lower well a significant (*p <* 0.05) effect of the functionalization on the TNF-α release was found. Values for P-MWCNTs were significantly (*p <* 0.05) higher than for MWCNT-COOH (Figure 4C).

IL-8 concentrations in the triple cell co-cultures were significantly (*p <* 0.05) increased for exposures to pre-coated MWCNTs (Figure 4D) in both the upper and the lower well. Except of the Curosurf pre-coating, no other influencing factors were observed to affect the IL-8 release. The ability of MWCNTs to adsorb

IL-8 to the surface, eliciting a false negative toxicity, was investigated in a previous study [44]. No significant IL-8 protein adsorption was observed.

## Discussion

As CNT production is increasing, there is an increased need to investigate potential adverse effects due to the possible human exposure. It is imperative that an understanding as to the underlying mechanisms and the identification of the key factors involved in any potential adverse effects are gained. In the present study, two important factors concerning the biological interaction of MWCNTs were investigated; (i) the coating of the MWCNTs surface with biomolecules from the pulmonary surfactant Curosurf, and (ii) the functionalization of the MWCNTs surface with positive and negative side groups. Both are modifications of the MWCNTs surface identity which are supposed to affect interaction of MWCNTs with their biological surroundings. Effects of both modifications are crucial in order to gain knowledge regarding the mechanisms associated with MWCNTs exposure to in the biological model but also to identify conditions (MWCNTs pre-coating, functionalization) with the lowest adverse effect levels.

In order to understand mechanisms that are underlying a cellular response following exposure, a mono and a co-culture in vitro approach were chosen. MDM monocultures were first exposed to MWCNTs under submersion exposure. These cells were used as a model for alveolar macrophages which are professional phagocytotic cells representing the first cellular “defense line” of the pulmonary immune system [45,46]. Primary macrophages derived from human blood monocytes were used, as they are known to retain their phenotypic differentiation more effectively when compared to macrophage cell lines [47]. Specific conditions of interest (concentrations, functionalization) were identified and then applied in an advanced 3D model of the human epithelial airway barrier [38,42,47].

### MWCNT uptake and cell morphology

After 24 h exposures of MDM, different MWCNTs were found intracellular both agglomerated and as single tubes. As different (functionalized and pre-coated) tubes were located in the cytoplasm but also in vesicles, different translocation mechanisms may be pertinent. For MWCNTs both have been described active processes, such as endocytosis or phagocytosis but also piercing of the cell membrane by single tubes or bundles [48,49]. However, it has to be emphasized that these processes strongly depend on the MWCNTs characteristics (such as christallinity, stiffness, length and agglomeration state [48]) and membrane piercing is mainly described for long and stiff CNTs, whereas short and entangled CNTs are preferentially enclosed by the cells [48]. Another mechanism which may explain MWCNTs in the cytoplasm is the endosomal escape. Mu et al. [49] showed (with the same MWCNTs as used in the present study), that tubes which were taken up by human embryonic kidney epithelial cells (HEK293) via endocytosis, were able to penetrate the endosomal membrane and escape into cytoplasm. None of the latter mechanisms can be excluded. However, a quantitative analysis of subcellular localization of CNTs is necessary to definitely elucidate this issue.

Visual observations clearly revealed that Curosurf coated MWCNTs were more often found in (intracellular) “bird's-nest”-like arrangements. This might be due to lipophilic surfactant components which foster the adhesion among MWCNTs as it was hypothesized in our previous cell free study [32] and other studies such as Kendall et al. [50] using carbon black. However, as dispersions with Curosurf pre-coated P-MWCNTs were clearly more stable compared to uncoated P-MWCNTs [32], the surface-active phospholipids may also foster deagglomeration and therefore counterbalance the former process.

In addition, binding of Curosurf compounds may directly affect uptake. For instance the surfactant protein A (SP-A) and a bovine surfactant preparation (Survanta) were found to increase the uptake of TiO_2_ particles into primary rat alveolar macrophages [51]. Konduru et al. [52] showed that SWCNT bound phosphatidylserine represents an “eat me” signal on their surface which facilitates the recognition and internalization by macrophages. As Curosurf contains phosphatidylserine, the pre-coating possibly also enhance uptake in the here presented MWCNT exposures. However, not only the binding of biomolecules, which was shown to increase cellular uptake (e.g. Konduru et al. [52] or Chin et al. [53]), may play an important role but also the altered dispersion from pulmonary surfactant may affect the biodistribution.

Despite a minor amount of MDM which showed apoptotic characteristics, the majority of cells didn’t present any structural impairment under different exposure conditions in both LM and TEM visual analyses. This finding is in accordance with the subsequently discussed biochemical endpoint analyses.

### Potential adverse effects of uncoated MWCNTs and the role of the functionalizations

Only minor adverse effects were found after exposures of MDM to uncoated MWCNTs. MDM showed an increased LDH release after MWCNT-NH_2_ exposures and the total intracellular glutathione content was decreased after 24 h for both MWCNT-NH_2_ and MWCNT-COOH exposures. The findings indicate a minor oxidative stress response in MDM after exposures to functionalized MWCNTs and they may also explain a slight increase in apoptosis after MWCNT-COOH exposures. Different surface characteristics and an improved dispersity (resulting in a larger surface area) compared to P-MWCNTs may explain these observations [27].

In the triple cell co-cultures (except of a remarkable increase in TNF-α release in the lower well after P-MWCNTs exposures), there were no indications for a possible inflammatory reaction after exposures to uncoated MWCNTs.

### Potential adverse effects of curosurf pre-coated MWCNTs

**Figure 5:**
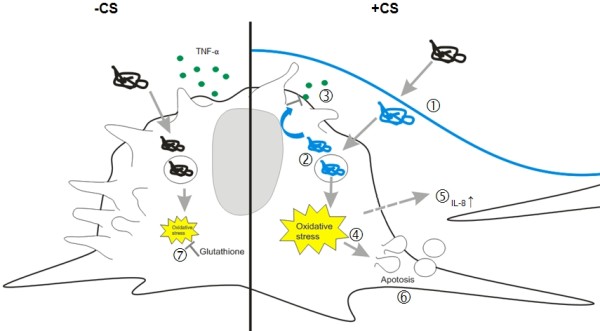
**Scheme of observed effects from MWCNTs, coated with Curosurf (+CS) or without (−CS) and the proposed underlying mechanisms.** Lipids and proteins of the surfactant bind to the MWCNTs and alter their surface characteristics (**1.**). Surfactant coated tubes are subsequently located in vesicles of MDM and free in the cytoplasm (**2.**). After 24 h a decrease in TNF-α release (**3.**) is observed which might be due to a down-regulation of the TNF-α mRNA by Curosurf compounds. An increase in ROS (**5.**) causes further an increase in IL-8 chemokine release in epithelial cells (**5.**) and induction of apoptosis (**6.**) in MDM. Uncoated MWCNTs which are present inside MDM after 24 h exposure, are inducing an intracellular glutathione depletion (**7.**).

A schematic interpretation of the main findings of Curosurf pre-coated MWCNTs is given in Figure 5.

Cytotoxicity (LDH release) and necrosis measurements didn’t show any indications of major cell membrane damage. In contrast, MDM exposures showed an induction of ROS after 1 h exposures to Curosurf pre-coated MWCNTs. This finding is consistent with literature (e.g. [54,55]), where an increased ROS production was reported after pre-coating of nano-objects with pulmonary surfactant “substitutes”. Herzog et al. [55] presumed an improved dispersion of SWCNTs after addition of dipalmitoylphosphatidylcholine (DPPC, the major component of lung surfactant) as a factor for the increase in oxidative stress. Furthermore, they suggest that there might be peroxidation of DPPC by free radicals which may explain the increase in H_2_O_2_ toxicity. An alteration of the SWCNTs surface chemistry is also claimed to affect the toxicity. By considering the binding of Curosurf compounds to different functionalized MWCNTs [32], such mechanisms may also play an important role in the here presented exposures.

No intracellular glutathione decrease was measured after 24 h for Curosurf pre-coated MWCNTs (in both MDM and triple cell co-cultures). These data indicate an early onset of ROS production and time-shifted reduction of cell antioxidant supply (i.e. the total intracellular glutathione) from pre-coated MWCNTs in MDM. Uptake may probably be faster for coated CNTs as it was shown by Konduru et al. [52] (see above).

Oxidative stress can result in the activation of signaling pathways via transcription factors such as NF-κB and AP-1, which then initiate the production of major pro-inflammatory mediators such as TNF-α or IL-8 [56,57].

Interestingly an increase in TNF-α release was not observed after 24 h. In contrast a reduction after exposures to Curosurf pre-coated MWCNTs (30 μg/ml) was observed in MDM. From different surfactant lipids and proteins, such as for the apolipoproteins, which were specifically detected on Curosurf pre-coated MWCNTs [32] it is known that they directly affect the inflammatory process [58].

An explanation therefore might be the suppression of the TNF-α response by phosphatidylserine (a surfactant lipid) as it was shown by Konduru et al. [52] with phosphatidylserine coated SWCNTs in RAW 264.7 macrophages. It is known that Curosurf down-regulates TNF-α mRNA in monocytes [59]. Thus, bound Curosurf compounds may become active after being transported into the cell by MWCNTs.

Oxidative stress and the redox state also regulate cell apoptosis via different pathways [60,61]. Such activation after increased oxidative stress may therefore also explain increased apoptosis in MDM after exposure to Curosurf pre-coated MWCNTs.

Lowest TNF-α release after exposures to 30 μg/ml Curosurf pre-coated MWCNT-COOH in triple cell co-cultures may be explained by the same mechanisms as it was proposed for MDM monocultures. In contrast to TNF-α release, IL-8 release was increased for exposures to Curosurf pre-coated MWCNTs in both the upper and the lower well of triple cell co-cultures. This might be due to interactions of epithelial cells with MDM (for which increased ROS was shown in monocultures) or from ROS of the epithelial cells.

Comparison of the different cell culture models

Tendencies for endpoints, which could be compared, such as cytotoxicity (LDH release), total intracellular glutathione or TNF-α release were very similar for both models. However, effects in triple cell co-cultures were in general attenuated compared to MDM. For instance a slight increase in cytotoxicity, which was observed in MDM after exposures to uncoated MWCNT-NH_2_ could not be observed in triple cell co-cultures. Similarly only a non-significant decrease of total intracellular glutathione levels was observed in the more complex model. It can be therefore suggested that the more realistic triple cell co-culture model has an attenuating effect on cellular responses due to the interplay of different cell types [62,63].

## Conclusions

In the present study, it was shown that MWCNTs pre-coated with Curosurf can penetrate cells of the airway epithelial barrier where the pre-coating evokes a mild increase in ROS, inflammatory chemokine release and apoptosis. However, these processes might be counterbalanced by a decrease of pro-inflammatory cytokines (TNF-α) which were observed after exposures to Curosurf pre-coated MWCNTs.

Bound compounds may not only affect the intracellular response to the MWCNTs but also uptake kinetics of MWCNTs might be altered and therefore probably also reaction times of cells to the MWCNTs.

Even if the effects of these different MWCNTs were relatively minor (and are further attenuated in more complex models), the differences between Curosurf pre-coated and bare MWCNTs should be considered for future studies. It will be of great interest to investigate the role of pulmonary surfactant in exposures to materials which have a higher adverse potential such as longer and more rigid MWCNTs [10,64,65]. But also for mechanistic studies using sub-lethal doses the pulmonary surfactant coating must be considered as key factor, as it may has consequences on lower levels such as cellular uptake, signaling or cell-cell communication, but also on higher levels i.e. in translocation trough tissue and in biodistribution.

## Methods

### MWCNT dispersions and cell exposure

MWCNTs were synthesized by chemical vapour deposition from Chengdu Carbon Nanomaterials R&D Center (Sichuan, China), functionalized and characterized as previously described [25,32].

MWCNTs were dispersed (1 mg/ml) in Curosurf 120 (Chiesi, Parma, Italy); a lipid-based porcine surfactant. Uncoated MWCNTs were directly dispersed in serum free cell culture media (1 mg/ml). Dispersions were sonicated with gentle agitation in a cooled sonicating water bath for 15 min. Subsequently, MWCNT stock solutions were diluted to final working concentrations of 0.3, 3 and 30 μg/ml in serum free cell culture medium (RPMI 1640 media containing 1% L-Glutamine and 1% Penicillin/Streptomycin). The highest concentrations (30 μg/ml) of the working solutions were adapted from Wick et al. [23] and Gangwal et al. [66]. Any higher concentration was chosen in order to avoid an overload situation. Dispersions of Curosurf pre-coated MWCNTs contained a maximum concentration of 3% free Curosurf. Thus an additional control exposure containing 3% Curosurf was always performed. For MDM exposures the cell culture medium in each well of the 6-well plate was replaced by 1 ml of the working dispersion. For triple cell co-cultures 1 ml of the working dispersion was applied to the upper well only.

### Human blood monocyte-derived macrophages (MDM)

Primary blood monocyte-derived macrophages (MDM) were isolated from human whole blood and cultured for 7 days as previously described [38,42].

### The triple cell co-culture model of the airway epithelial barrier

An in vitro triple cell co-culture model of the human epithelial airway barrier consisting of human epithelial cells (16HBE14o cell-line), human blood monocyte-derived dendritic cells (MDDC) and MDM, was cultured as previously described [38]. Briefly, 16HBE14o cells were grown to confluence on a microporous membrane. For composing the triple cell co-cultures MDM were added to the upper side of the transwell membrane and MDDC to the basal side [38,42].

### Lactate dehydrogenase (LDH) release

After 24 h of exposure the extracellular LDH was measured using a Cytotoxicity Detection Kit from Roche Applied Science (Mannheim, Germany) according to the supplier's manual. As a positive control, cells were treated with 0.2% Triton X100 in PBS for 24 h. (n = 3 for MDM, n = 5 for triple cell co-cultures).

### Detections of reactive oxygen species

MDM were loaded with H_2_DCF-DA (Invitrogen, Carlsbad, USA) for 1 h, subsequently washed with HBSS (Invitrogen, Carlsbad, USA) and exposed for 1 h to the panel of different MWCNTs. The fluorescent intensity was then quantified by an LSR II flow cytometer (BD Biosciences, Franklin Lakes, USA). The nitrite oxide donor 3-morpholinosydnonimine (Sin-1) (Sigma-Aldrich, St. Louis, USA) was used at 5 μM in HBSS as a positive control. Data was analysed using FlowJo (Tree Star, Ashland, USA). (n = 3).

### Intracellular glutathione quantification

After removing the supernatant from the 24 h exposures, the intracellular glutathione levels were quantified using a Glutathione Assay Kit (Cayman chemical, Michigan, USA) according to the supplier's manual. Quantified amounts of glutathione were normalized to total protein contents, which were measured by a Pierce BCA Protein Assay Kit (Thermo Fisher Scientific, Rockford, USA). As a positive control, tert-Butyl hydroperoxide (Sigma-Aldrich, St. Louis, USA) was used at100 μM for MDM and 100 mM for the triple cell co-cultures for 24 h. (n = 3 for MDM, n = 5 for triple cell co-cultures).

### Cytokine quantification

Tumor necrosis factor (TNF-α) and Interleukin-8 (IL-8) concentrations were quantified by a commercially available DuoSet ELISA Development Kit (R&D Systems, Oxon, UK) according to the manufacturer's recommendations.

Lipopolysaccharide (LPS) (Sigma-Aldrich, St. Louis, USA) at 30 μg/ml was used as a positive control for both MDM and co-culture exposures. (n = 4 for MDM, n = 5 for triple cell co-cultures).

### Cell death

The cell death protocol was adapted from Kieninger et al. [67]: After 24 h exposure, MDM were washed twice in cold PBS. Briefly, cells were then harvested by pipetting and resuspended in binding buffer (10 mM EPES/NaOH, pH 7.4, 140 mM NaCl, 2.5 mM CaCl_2_). Staining was strictly performed on ice throughout the entire procedure. Cell samples were treated with Annexin-V (1 μg/ml) (BD Biosciences, Franklin Lakes, USA), and incubated for 10 min. Cells were washed twice and incubated with the Streptavidin-conjugated Allophycocyanin secondary antibody (eBioscience, San Diego, USA) for 10 min. After 3 washes, 250 μl of cold binding buffer was added to the tubes immediately before analysis with an LSR II flow cytometer (BD Biosciences, Franklin Lakes, USA). Propidium iodide (2.5 μg/ml) (BD Biosciences, Franklin Lakes, USA) was added to the samples. Cells which were frozen for 30 min at −80°C or fixed with formaldehyde 4% for 30 min at room temperature, served as positive controls for necrosis and apoptosis, respectively. Data was analysed using FlowJo (Tree Star, Ashland, USA). (n = 5).

### Light microscopy

Light micrographs were taken with a Leica DFC425 C digital camera on a Leica DMI 4000 B microscope.

### Transmission electron microscopy

Cells were pelleted and sucked up into a capillary tube (Leica Microsystems, Wetzlar, Germany), fixed with 3% glutaraldehyde and postfixed with 2% osmium tetroxide. After dehydration through graded ethanol series followed by acetone cells were embedded in Epon resin (Sigma-Aldrich, St. Louis, USA). Ultrathin sections were contrasted with 2% uranyl acetate and lead citrate [68]. Analyses were performed on a Zeiss (Oberkochen, Germany) EM 900 at 80 kV.

### Statistical analysis

Residues were calculated for all data and tested for normal distribution using a Kolmogorov–Smirnov test. The influence of independent variables (Curosurf pre-coating, concentration, functionalization) was tested with an analysis of variance (ANOVA). Bonferroni t-tests were carried out to compare subgroups against each other. All analyses were performed using the statistical software SPSS V.18 (Dynelytics, Zurich, Switzerland).

## Competing interests

The authors declare that they have no competing financial interest.

## Authors' contributions

MG participated in the design of the study, carried out the experimental work and drafted the manuscript. PW and MJDC were involved in planning the design of the study, accompanied the experimental work intellectually and made substantial contributions to the analysis and interpretation of the data. FB was involved in the flow cytometry analyses and in the interpretation of data. LD conducted the TEM sample preparation and analyses and was involved in the visual interpretations. BY designed the functionalized MWCNTs and has been involved in revising the manuscript. HFK and PG were involved in the planning of the study and in data interpretation. BR was the project leader; she was involved in planning the design of the study, has intellectually accompanied the experimental work, made substantial contributions to the analysis and interpretation of the data, has been involved in revising the manuscript critically for important intellectual content and has given final approval of the version to be published. All authors read and approved the final draft.

## Supplementary Material

Additional file 1 **Table S1.** Characterization of the different functionalized MWCNTs. Click here for file

Additional file 2 **Statistical analysis: Analysis of variance (ANOVA) and Bonferroni post-hoc tests.** The influence of 3 the independent variables concentration, functionalization and Curosurf pre-coating were tested on different endpoints using an ANOVA. The *p*-values (*=*p <* 0.05, **= *p <* 0.01) are shown. As only MWCNT-COOH were pre-coated for the triple cell co-culture experiments no *p*-values are shown in the corresponding section for interactions with the functionalization. §: A Bonferroni *post-hoc* test shows significant (*p <* 0.05) differences between 0.3 μg/ml and 30 μg/ml and between 3 μg/ml and 30μg/ml. §§: A Bonferroni *post-hoc* test shows a significant (*p <* 0.05) difference between P-MWCNT and MWCNT-NH2. §§§: A Bonferroni *post-hoc* test shows a significant (*p <* 0.05) difference between P-MWCNT and MWCNT-COOH. §§§§: A Bonferroni *post-hoc* test shows a significant (*p <* 0.05) difference between P-MWCNT and MWCNT-COOH. Abbreviations for different endpoints: LDH lactate dehydrogenase, ROS reactive oxygen species, GSH intracellular glutathione, TNF-α, IL-8 interleukin 8. Click here for file
