# Supplementary material for: Pulmonary surfactant coating of multi-walled carbon nanotubes (MWCNTs) influences their oxidative and pro-inflammatory potential in vitro
Source: Part Fibre Toxicol. 2012 May 24;9:17. doi: 10.1186/1743-8977-9-17 (PMC3496593; doi:10.1186/1743-8977-9-17)
Supplement: Additional file 1 — Table S1. Characterization of the different functionalized MWCNTs. [file 1743-8977-9-17-S1.doc]

## Additional file 1: Table S1 Characterization of the different functionalized MWCNTs

|  | **P-MWCNTs** | **MWCNT- NH_2_** | **MWCNT- COOH** | References / Technique |
| --- | --- | --- | --- | --- |
| Length | 500 to >2000 nm | 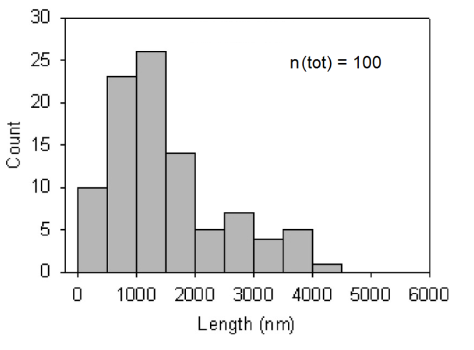 | 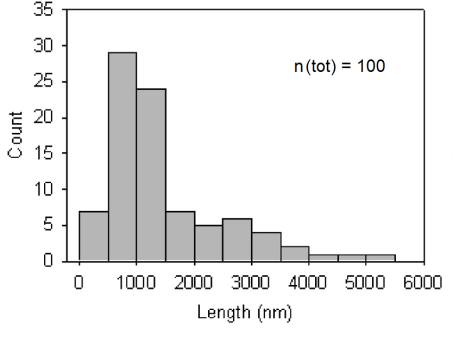 | [32,49] |
| Diameter [nm] | 40-50 | | | TEM |
| Specific surface area [m^2^/g] | 500 | | | Per manufacturer (Chengdu Carbon Nanomaterials R&D Center). 2009 |
| Metal impurities | Fe (0.3%), Al (0.02%), Cr (0.002%), other 20 metals (<0.00008%) | | | ICP-MS |
| Zeta-potential in H_2_O [mV] | -2 | +26 | -57 | [32] |
| Zeta-potential in Curosurf [mV] | -63 | -50 | -56 | [32] |
| Number of modifi-cations  [/nm^2^] | - | 5 | | Calculated from experimental loading and surface area |
| TEM micro-graphs MWCNTs in H_2_O  (Scalebar 500 nm) | 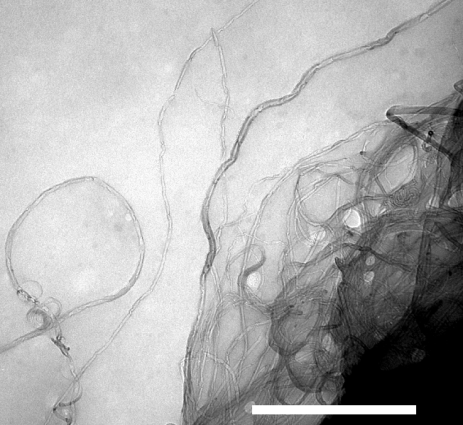 | 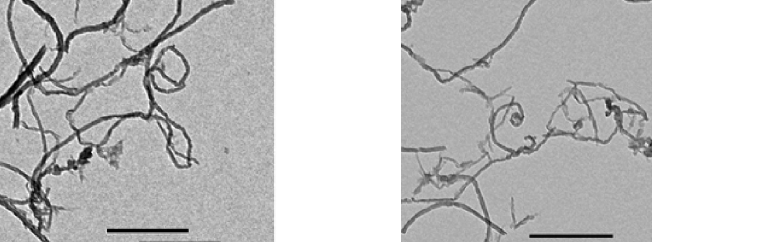 | 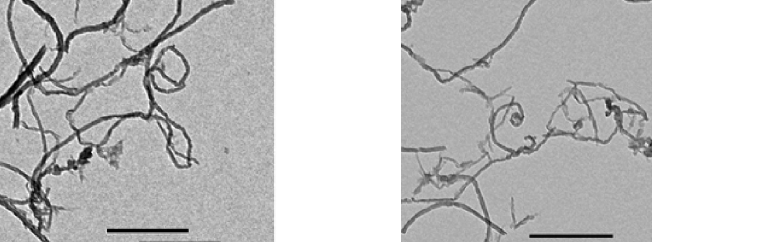 | Central and right picture from Mu et al. [49] (left picture from our lab) |
| MWCNTs in medium  24h after sonication (200µg/ml stock) | 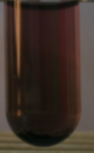 | 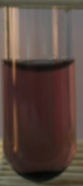 | 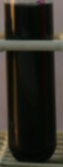 |  |
| Agglomer-ation/  Aggregation status | Precipitation of agglomerates in aqueous dispersions | Stable dispersion in aqueous media after sonication. Some precipitation can be observed after 24h | Stable dispersion in aqueous media |  |
|  | MWCNT dispersions are stable in Curosurf. | | | |
