# Supplementary material for: Pulmonary surfactant coating of multi-walled carbon nanotubes (MWCNTs) influences their oxidative and pro-inflammatory potential in vitro
Source: Part Fibre Toxicol. 2012 May 24;9:17. doi: 10.1186/1743-8977-9-17 (PMC3496593; doi:10.1186/1743-8977-9-17)
Supplement: Additional file 2 — Statistical analysis: Analysis of variance (ANOVA) and Bonferroni post-hoc tests. The influence of 3 the independent variables concentration, functionalization and Curosurf pre-coating were tested on different endpoints using an ANOVA. The p-values (*=p < 0.05, **= p < 0.01) are shown. As only MWCNT-COOH were pre-coated for the triple cell co-culture experiments no p-values are shown in the corresponding section for interactions with the functionalization. §: A Bonferroni post-hoc test shows significant (p < 0.05) differences between 0.3 μg/ml and 30 μg/ml and between 3 μg/ml and 30μg/ml. §§: A Bonferroni post-hoc test shows a significant (p < 0.05) difference between P-MWCNT and MWCNT-NH2. §§§: A Bonferroni post-hoc test shows a significant (p < 0.05) difference between P-MWCNT and MWCNT-COOH. §§§§: A Bonferroni post-hoc test shows a significant (p < 0.05) difference between P-MWCNT and MWCNT-COOH. Abbreviations for different endpoints: LDH lactate dehydrogenase, ROS reactive oxygen species, GSH intracellular glutathione, TNF-α, IL-8 interleukin 8. [file 1743-8977-9-17-S2.pdf]

	Independent variables						
	Concentration	Functionalization	Curosurf pre-coating	Concentration * Functionalization (interactions)	Concentration * Pre-coating (interactions)	Functionalization * Pre-coating (interactions)	Concentration * Functionalization * Pre-coating (interactions)
				**MDM**			
LDH	0.23	0.15	***0.01**	0.67	0.62	0.37	1.00
ROS	0.16	0.29	****0.00**	0.99	0.09	0.96	0.63
GSH	0.66	0.07	***0.01**	0.55	0.64	0.56	0.73
TNF-α	***§0.01**	***§§0.04**	0.86	0.63	***0.01**	0.74	0.59
Necrosis	0.74	0.47	***0.01**	0.30	0.94	0.82	0.31
Apoptosis	***0.04**	**§§§*0.03**	***0.03**	0.86	0.41	0.83	0.85
				**Triple cell co-cultures (upper well)**			
LDH	0.82	0.25	0.69	0.63	0.54	n.a.	n.a.
TNF-α	***0.01**	0.14	0.22	***0.04**	0.18	n.a.	n.a.
IL-8	0.2	0.35	***0.01**	0.5	0.76	n.a.	n.a.
				**Triple cell co-cultures (lower well)**			
LDH	0.76	0.55	0.27	0.2	***0.04**	n.a.	n.a.
TNF-α	0.72	***§§§§0.03**	0.78	0.41	0.26	n.a.	n.a.
IL-8	0.23	0.36	***0.01**	0.8	0.19	n.a.	n.a.
GSH	0.13	0.45	0.06	1.00	0.63	n.a.	n.a.

Statistical analysis: Analysis of variance (ANOVA) and Bonferroni post-hoc tests.

The influence of 3 the independent variables concentration, functionalization and Curosurf pre-coating were tested on different endpoints using an ANOVA. The *p*-values (*=$p < 0.05$, **= $p < 0.01$) are shown. As only MWCNT-COOH were pre-coated for the triple cell co-culture experiments no *p*-values are shown in the corresponding section for interactions with the functionalization. §: A Bonferroni *post-hoc* test shows significant ($p < 0.05$) differences between 0.3 µg/ml and 30 µg/ml and between 3 µg/ml and 30µg/ml. §§: A Bonferroni *post-hoc* test shows a significant ($p < 0.05$) difference between P-MWCNT and MWCNT-NH_2. §§§: A Bonferroni *post-hoc* test shows a significant ($p < 0.05$) difference between P-MWCNT and MWCNT-COOH. §§§§: A Bonferroni *post-hoc* test shows a significant ($p < 0.05$) difference between P-MWCNT and MWCNT-COOH. Abbreviations for different endpoints: LDH lactate dehydrogenase, ROS reactive oxygen species, GSH intracellular glutathione, TNF-α, IL-8 interleukin 8.
